# Characterising Post-mortem Bacterial Translocation Under Clinical Conditions Using 16S rRNA Gene Sequencing in Two Animal Models

**DOI:** 10.3389/fmicb.2021.649312

**Published:** 2021-05-31

**Authors:** Lily Gates, Nigel J. Klein, Neil J. Sebire, Dagmar G. Alber

**Affiliations:** ^1^Department of Infection, Immunity and Inflammation, University College London Institute of Child Health, London, United Kingdom; ^2^Histopathology, Great Ormond Street Hospital, London, United Kingdom

**Keywords:** post-mortem, bacterial translocation, 16S sequencing, SUDI, SIDS

## Abstract

Sudden unexpected death in infancy (SUDI) is the sudden and unexpected death of an apparently healthy infant occurring within the first year of life where the cause is not immediately obvious. It is believed that a proportion of unexplained infant deaths are due to an infection that remains undiagnosed. The interpretation of post-mortem microbiology results is difficult due to the potential false-positives, a source of which is post-mortem bacterial translocation. Post-mortem bacterial translocation is the spread of viable bacteria from highly colonised sites to extra-intestinal tissues. We hypothesise that although post-mortem bacterial translocation occurs, when carcasses are kept under controlled routine clinical conditions it is not extensive and can be defined using 16S rRNA gene sequencing. With this knowledge, implementation of the 16S rRNA gene sequencing technique into routine clinical diagnostics would allow a more reliable retrospective diagnosis of ante-mortem infection. Therefore, the aim of this study was to establish the extent of post-mortem bacterial translocation in two animal models to establish a baseline sequencing signal for the post-mortem process. To do this we used 16S rRNA gene sequencing in two animal models over a 2 week period to investigate (1) the bacterial community succession in regions of high bacterial colonisation, and (2) the bacterial presence in visceral tissues routinely sampled during autopsy for microbiological investigation. We found no evidence for significant and consistent post-mortem bacterial translocation in the mouse model. Although bacteria were detected in tissues in the piglet model, we did not find significant and consistent evidence for post-mortem bacterial translocation from the gastrointestinal tract or nasal cavity. These data do not support the concept of significant post-mortem translocation as part of the normal post-mortem process.

## Introduction

Sudden unexpected death in infancy (SUDI) is the sudden death of an apparently healthy infant occurring within the first year of life where the cause is not immediately obvious. SUDI is the leading cause of infant mortality in the developed world (Spencer and Logan, 2004; Ndu, 2016). Despite extensive post-mortem (PM) investigations into the cause of death, around 40% of SUDI cases remain unexplained and are subsequently registered as Sudden Infant Death Syndrome (SIDS) (Office for National Statistics, 2017). Infection is one of the leading causes of SUDI (Weber et al., 2008; Pryce et al., 2011b; Goldwater, 2017) and it has been hypothesised that infection is in fact responsible for a larger proportion of SUDI, but these infections remain undetected due to limited diagnostic power using current methodologies (Weber et al., 2008; Goldwater, 2009, 2017).

In diagnostic laboratories worldwide, classical bacterial culture is the gold-standard method used to identify potential causative organisms as part of routine clinical autopsy investigation (Kennedy, 2016). However, these methods are associated with many limitations, such as an increased likelihood of identifying readily culturable bacteria (Yu et al., 2019), the inability to culture all bacterial species under laboratory conditions (Hiergeist et al., 2015; Overmann et al., 2017), long incubation periods (Ombelet et al., 2019), skilled persons or high-cost equipment for bacterial identification, and the curation of only qualitative results (Rhoads et al., 2012). The interpretation of PM microbiology results generated in this way proves difficult, particularly in cases of SUDI where histological findings are often unclear (Pryce et al., 2011b; Palmiere et al., 2016).

In recent years, these caveats have encouraged the use of alternative molecular techniques, such as quantitative PCR (qPCR) targeting known pathogenic bacteria, or the sequencing of the bacterial 16S rRNA gene. The 16S rRNA gene is present in all bacterial species and each has a unique sequence. These sequences act as a bacterial fingerprint to enable bacterial identification by comparison to curated 16S reference databases, such as Greengenes, SILVA, and RDP (Wang et al., 2007; McDonald et al., 2012; Yilmaz et al., 2014). This method allows for a more detailed, unbiased determination of the bacteria present within tissues. This technique has been implemented throughout forensic literature, with particular interest placed on the use of the PM microbiome, commonly referred to as the thanatomicrobiome, as a microbial clock to determine the post-mortem interval (PMI) in advanced phases of decomposition (Javan et al., 2019; Roy et al., 2021). Still, these methods have not been widely incorporated into routine PM investigation, as their performance and interpretation remain uncertain in this setting (Fernández-Rodríguez et al., 2019).

Apparent false-positive PM microbiology results can arise due to the natural putrefaction process of the body or contamination during sample collection at autopsy (Morris et al., 2006; Riedel, 2014; Mesli et al., 2017). When positive cultures arise from visceral tissues that are believed to be sterile in life, it is often difficult to determine the relevance of such results, certainly without histological evidence of infection (Goldwater, 2009; Bryant and Sebire, 2018). PM bacterial translocation is defined as the migration of viable bacteria from highly colonised bodily sites, such as the gastrointestinal (GI) tract, to extra-intestinal tissues following death (Mesli et al., 2017).

Few studies have investigated the phenomenon of PM bacterial translocation, and those that have, typically investigate bacterial invasion under ambient conditions. Heimesaat et al. (2012) investigated PM translocation in a mouse model kept under ambient conditions using conventional bacterial culture. Results from this study showed that extra-intestinal sites including cardiac blood, kidney, liver, mesenteric lymph nodes, and spleen were invaded by intestinal bacteria as soon as 5 minutes PM (Heimesaat et al., 2012). Another study tracked fluorescently labelled *Staphylococcus aureus* following intranasal inoculation of a mouse model and found that these bacteria could be detected in all organs 1 hour PM (Burcham et al., 2016). Again, this animal model was kept under ambient conditions following sacrifice.

There is a current lack of understanding of the PM bacterial translocation process in a clinical setting where bodies are refrigerated between death and autopsy (Morris et al., 2006; Weber et al., 2010; Palmiere et al., 2016; Mesli et al., 2017). This lack of knowledge presents a greater problem when interpreting PM microbiological results during clinical autopsy and poses the question, ‘*is a positive result due to natural decomposition and translocation of bacterial flora or a sign of fulminant ante-mortem infection?*’. Interpretation of results requires professionals to determine whether a positive finding is a contaminant or causative of the fatal event (Balzan et al., 2007). Unlike contamination during sampling, putrefaction is a natural process that can be limited, but not completely controlled (Morris et al., 2006). Routine procedures, such as refrigeration of the body soon after death aims to limit decomposition, but the extent to which this prevents bacterial translocation has yet to be studied.

To our knowledge, PM bacterial translocation has not yet been characterised under conditions mimicking standard clinical practice using contemporary molecular approaches. Essential to the interpretation of PM microbiological results of SUDI, is a clear understanding of the natural bacterial translocation that occurs PM in this clinical setting. We hypothesise that although PM bacterial translocation occurs, it is not extensive and can be defined using 16S rRNA gene sequencing. With this knowledge, implementation of this technique into routine clinical diagnostics would then enable a more reliable retrospective diagnosis of ante-mortem infection. Therefore, the aim of this study was to establish the extent of PM bacterial translocation in two animal models to establish a baseline sequencing signal for the PM process.

We report the use of 16S rRNA gene sequencing in two separate animal models; a mouse model for ease of sampling, followed by a piglet model given its anatomical similarities to humans and ability to perform repeated sampling, to investigate bacterial presence in visceral tissues routinely sampled during autopsy for microbiological investigation. Using these animal models allowed for controlled conditions and multiple sampling time points over the study starting from time of death. We aimed to investigate (1) the bacterial community succession in regions of high bacterial colonisation, and (2) the translocation of these microbes to extra-intestinal tissue over a 2-week period PM.

## Materials and Methods

### Sample Acquisition

Nine-week old C57BL/6 female mice (*n* = 15) that had been housed for 6 days to allow for acclimatisation and standardisation of the microbiota for another study were culled on day 0 according to Schedule 1 of the Animals (Scientific Procedures) Act 1986 and carcasses were donated to this study. Culled mice were placed into airtight freezer bags and kept on wet ice until the first sample was collected, no longer than 30 minutes PM. Tissue and blood samples were collected from one mouse per cage (C) on the following days (D) PM: D0, D3, D7, D10, and D14 (three mice sampled per day) in a sterile manner to limit contamination. Tissue samples were collected in the following order: cardiac blood, heart, lung, liver, spleen, and the lower GI tract. Mice were stored in airtight freezer bags at 4°C until sampling.

Three 1-month-old Welsh piglet carcasses were obtained from the Royal Veterinary College, London (*n* = 1) and a local farm (*n* = 2). The piglets used in this study were of a pre-weaning age. Sampling was initiated no more than 3 hours PM. Samples were obtained using sterile 16-gauge Quick-Core Biopsy Needles (Cook Medical, Ireland) on D0, D3, D7, D10, and D14 PM. On D14 PM, carcasses from the local farm were dissected using sterile scalpels. Nasal (left and right nares) and rectal swabs were obtained using polyurethane cellular foam dry swabs (MWE Medical Wire). Samples were immediately snap frozen and stored at −80°C until further analysis. Tissue type confirmation was performed using tissue-specific reverse transcriptase PCR as described in the Supplementary Methods 1. Piglet carcasses were stored in infant body bags at 4°C in between sampling time points.

### DNA Extraction and Sequencing

DNA was extracted using the QIAamp DNA Mini Kit (Qiagen) as per the manufacturers protocol with an initial lysing step using Ribolysing Matrix B (MP Biomedicals) and bead-beating for 1 minute at 50 oscillations/second. DNA templates were amplified for sequencing using the Taq PCR Core Kit (Qiagen) and region-specific primers targeting the V3-V4 region of the 16S rRNA gene with adapters and indexes attached as previously described (Rosser et al., 2020). This method has been previously validated in-house using spike-in experiments whereby Gram-positive and Gram-negative bacteria were added to various tissue types to ensure recovery of the 16S rRNA gene (data not published). A Microbial Community Standard (Zymo Research) of known bacterial composition was used to assess bias and sequencing error rates. A post-PCR concentration of <0.5 ng/μL was considered negative. Tissue samples were amplified thrice before being considered negative. Positive samples were normalised to the desired library concentration using nuclease-free water, pooled, and sequenced on the MiSeq platform using a 500-v2 cartridge (Illumina).

### Quantitative qPCR for Enterobacteriaceae and Enterococcus

See Supplementary Methods 2, 3.

### Sequencing Data Processing and Statistical Analysis

Sequencing data was demultiplexed on the MiSeq platform. Paired-end reads were merged using FLASH (version 1.2.11) (Magoč and Salzberg, 2011) and quality filtered using VSEARCH. Taxonomic classification was carried out on the Mothur platform (version 1.44.0) (Schloss et al., 2009) using the RDP reference database with a similarity cut-off of 97%. Data was analysed using R Studio (version 1.2.5001) (RStudio Team, 2020). Samples with sequencing reads within two standard deviations from the mean number of reads within the extraction control samples were removed. The mock community was assessed for any unexpected spurious OTUs and abundances were used as a cut-off for false-positive sequence generation.

Statistical analysis was performed using R (version 3.6.3) (R Core Team, 2019) on R Studio (RStudio Team, 2020). Rarefaction curves were generated to assess species richness and appropriate random subsampling thresholds using the *vegan* package (Oksanen et al., 2019). Mouse GI tract samples were randomly subsampled at 20,000 reads. Piglet nasal and rectal swabs were randomly subsampled at 100,000 and 14,000 reads, respectively. Two samples (nasal swab P3_D10_right and rectal swab P2_D3) were excluded from downstream analysis due to low quality sequencing reads. Differences in the total number of generated sequencing reads that passed quality filtering were statistically compared between sample type and organism sampled using an independent *t*-test. Piglet tissues were subsampled to 500 reads prior to beta-diversity calculation. Changes in community structure in the piglet tissues (beta-diversity) was assessed with a non-metric multi-dimensional scaling (NMDS) plot based on Bray-Curtis dissimilarities between the samples. The effects of sampling day on bacterial community changes were measured by performing a permutational multivariate analysis of variance (PERMANOVA) test on Bray-Curtis dissimilarity measures using the *phyloseq* package (McMurdie and Holmes, 2013). A Kruskal-Wallis test by ranks was performed on communities at different time points to compare taxonomic differences at family level classification using the *stats* package (R Core Team, 2019). For statistical tests a *P* < 0.05 was considered significant. Figures were generated using the *ggplot2* package (Hadley, 2016) and Graphpad Prism (version 5) (Motulsky, 2007).

## Results

### Mouse Gut Microbiota Is Relatively Stable Over the Study Period

**FIGURE 1:**
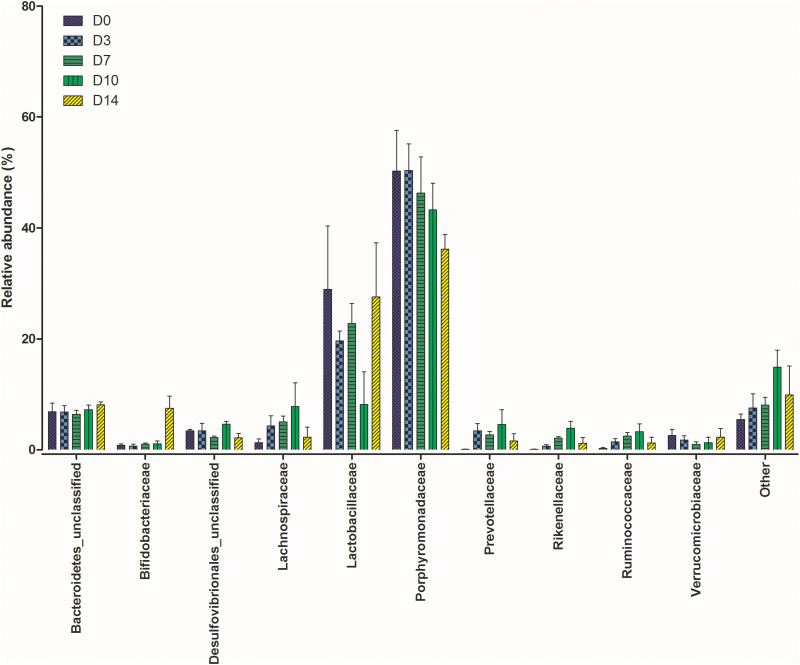
Shifts in the relative abundance of the top 10 bacterial families present within the mouse gastrointestinal (GI) tract samples collected at five time points post mortem.

The gut microbiome in all mice sampled at each time point were similar as expected due to their inbred nature and controlled housing environment and diet prior to this study. Based on the Bray-Curtis distances calculated for each community, there was no significant difference between communities from each mouse (*P* = 0.07). Figure 1 shows the relative abundance of the top 10 bacterial families at each time point (plots for individual samples are available in Supplementary Figure 1).

The calculated beta-diversity at each time point was compared to that of D0 to track microbiota changes over time. The first 7 days PM showed no significant changes in the gut microbiome (*P* = 0.096). Significant changes were, however, observed in the communities on D10 (*P* = 0.007) and D14 (*P* = 0.037) when compared to D0. To investigate bacterial families responsible for these differences a Kruskal-Wallis by rank test was performed. Dominant families did not experience any changes in rank over the study period. Significant differences were observed in Bifidobacteriaceae (*P* = 0.013) and Rikenellaceae (*P* = 0.041). At each time point from D0 to D10 Bifidobacteriaceae represented less than 1% of the total bacterial community. This was followed by a sharp increase on D14 where their relative abundance increased to 7.2%. Rikenellaceae was also present at less than 1% in the starting community. There was a steady increase to D10 where the relative abundance peaked at an average of 3.9%, which decreased again at D14 where it represented just 0.6% of the community.

### Post-mortem Bacterial Translocation Could Not Be Detected in the Mouse Model

Of the 75 mouse tissue samples collected, only 4 were considered as positive for the 16S rRNA gene using the quality filtering threshold described in the methods section (positive samples are detailed in Supplementary Table 1). The positive tissue samples had an average of 3,267 sequencing reads (range 642–10,839). The number of sequencing reads in the tissue samples were significantly lower than those in the GI tract samples (*P* = 0.004). The number of successfully sequenced samples did not increase with time PM.

Operational taxonomic units (OTUs) detected in each tissue sample were cross-checked with OTUs identified in the GI tract to assess the potential translocation of bacteria from this site. Two of the four samples (C1_D3_Liver and C2_D14_Blood) did not share any OTUs with the GI tract samples collected from the same mouse at the same time point. Sample C2_D3_Liver shared all OTUs with the corresponding GI tract sample and contained bacterial families including Enterobacteriaceae, Lactobacilliaceae, and Porphyromonadaceae. These families were of high abundance in the GI tract. Sample C2_D10_Heart shared 4 of 11 OTUs identified with its corresponding GI tract sample.

Three of the four tissue samples were dominated by either Enterobacteriaceae or Enterococcus, which represented >45% of each sample. To identify whether these were truly positive reads, a specific qPCR was performed. These bacteria were identified in three gut samples from cage 2, at low relative abundances between 5 and 11%. All tissues were negative for Enterococcus. Two of the three tissues were positive for Enterobacteriaceae, but generated a very late signal with a cyclic threshold (CT) value >34 compared to the positive controls which gave an average CT value of 27.

### No Trends Observed Amongst Piglets in the Rectal Swabs

**FIGURE 2:**
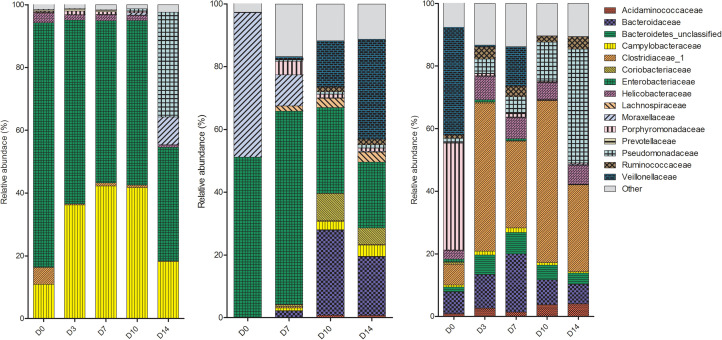
Shifts in the relative abundance of the top 10 bacterial families in piglet rectal swabs collected from piglet 1, piglet 2, and piglet 3 (left to right). The x-axis describes the day on which the sample was collected (D0, D3, D7, D10 or D14).

The microbes within the rectal cavities were significantly different in each piglet as expected due to differences in genetics, diet and environmental conditions (*P* = 0.018). The relative abundance of bacterial families in each piglet are shown in Figure 2. The rectal swab communities progressed in a different manner over the 2-week period in each piglet. In P1, the bacterial community in the rectal cavity remained relatively stable for the first 10 days PM. On D14, an increase in Pseudomonadaceae and Moraxellaceae was observed. The dominant families in the rectal cavities in P2 and P3 included Enterobacteriaceae and Moraxellaceae. As time progressed the Bacteroidaceae and Veillonellaceae families increased in relative abundance.

### Nasal Swabs Were Progressively Dominated by *Pseudomonas* spp.

**FIGURE 3:**
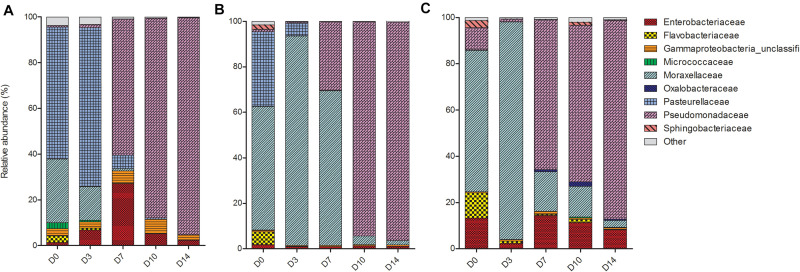
Shifts in the relative abundance of the top 10 bacterial families in piglet nasal swabs collected from **(A)** piglet 1, **(B)** piglet 2, and **(C)** piglet 3. The x-axis describes the day on which the sample was collected (D0, D3, D7, D10, D14).

Despite differences in early communities, nasal swab communities from all three piglets progressed in a similar fashion (Figure 3). On D0, the main bacterial families present in the nasal swabs were Enterobacteriaceae, Flavobacteriaceae, Moraxellaceae, and Pasteruellaceae. From D3 to D14, communities in different piglets began to converge and at both D10 and D14 there was no significant difference between all three piglets (*P* = 0.33). The convergence was largely due to the increase of Pseudomonadaceae, which began to emerge on D7 and increased in abundance until the end of the study period. On D14 PM, Pseudomonadaceae represented >90% of bacteria within the nasal cavity of each piglet.

### Potential PM Bacterial Translocation Is Observed in the Piglet Model

As the piglet tissue samples were obtained using needle biopsies in a blind fashion the tissue type was confirmed using a reverse-transcriptase qPCR (successful sampling shown in Supplementary Table 2). The success rate of sampling the heart, the liver, and the lung in P1, P2 and P3 was 87% (13/15), 93% (14/15), and 87% (13/15), respectively (total of 40 tissue sample successfully collected). Sequencing data were obtained for 29 of these 40 tissue samples. Sequencing reads in the piglet tissues were higher than those obtained from the mouse tissues with an average of 30,960 reads from tissues across all three piglets. These read numbers were, however, significantly lower than those found in the nasal and rectal swabs (*P* = 0.0001). Variation amongst the reads obtained from each piglet was observed, but there were no associations with sampling day or tissue type.

**FIGURE 4:**
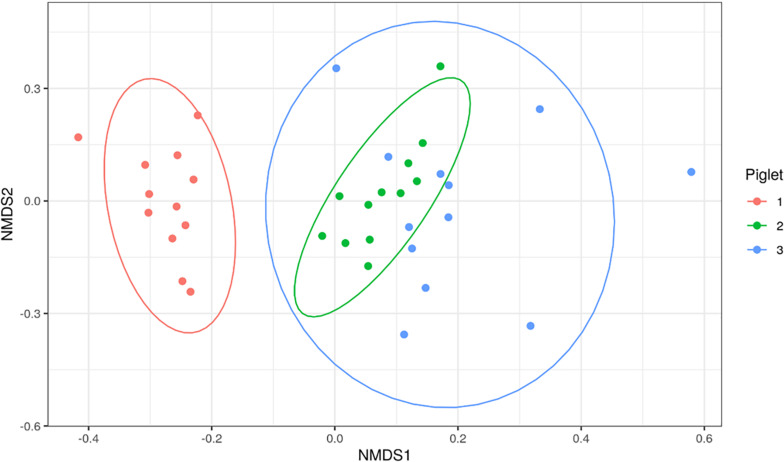
Non-metric multi-dimensional scaling (NMDS) plot based on Bray-Curtis dissimilarity index of piglet tissue samples. Each point represents an individual piglet tissue sample. Ellipses are based on 95% confidence intervals. Colours represent the piglet from which the sample was collected.

Bacterial families identified in the piglet tissues were assessed using beta-diversity measures. Figure 4 shows an NMDS plot based on calculated Bray-Curtis distances of beta-diversity. Each point on the NMDS plot represents a single tissue sample. This plot shows that the bacterial communities cluster based on the piglet they were sampled from rather than time-point or sample type. The dominant bacterial families were shared amongst tissues from the same piglet. Tissues collected from P1 were mainly dominated by Clostridiaceae_1 (7/10), P2 tissues were dominated by Moraxellaceae (4/10) and P3 tissues by Lactobacilliaceae (4/9).

As the aim of this study was to determine whether translocation occurs from sites of high colonisation to otherwise sterile tissues, OTUs present within the tissues were cross-checked with those identified in nasal and rectal swabs from the same piglet to identify if this was a possible source of translocation. Of the 29 sequenced samples, only 10/29 (34%) shared an OTU that was present at relative abundances >1% in the rectal swabs (Supplementary Figure 2A). Of the 10 samples that shared at least one OTU with the rectal swabs, the dominant OTU in 3/10 (30%) of the tissue samples was found in the rectal swabs. In comparison to the nasal swabs, 8/29 (28%) of the tissue samples shared at least one OTU present >1% in the nasal cavity, of which 1/8 (13%) shared their dominant OTU.

## Discussion

The aim of this study was to establish the extent of PM bacterial translocation in two animal models to establish a baseline sequencing signal for the PM process. This study provides insight into the community succession within the mouse GI tract, and the piglet nasal and rectal cavities. Our results also demonstrate that significant and consistent PM bacterial translocation was not observed in the mouse model, shown by broad-range 16S rRNA gene sequencing and confirmed with more specific, bacteria-targeted qPCR. Although bacteria were detected amongst tissues in the piglet model, we did not find evidence for true bacterial translocation from the GI tract or nasal cavity using these techniques. These data do therefore not support the concept of significant PM translocation as part of the normal PM process and demonstrate that the 16S rRNA gene sequencing technique could prove useful in diagnosis of infection PM.

Though the mouse gut microbiome was relatively stable over the first 7 days, significant changes were observed in Bifidobacteriaceae and Ruminococcaceae families at latter time points, increasing in abundance over time. These bacteria are known anaerobes, which explains their proliferation at a time when oxygen sources are low, and nutrients limited. The very slight changes in the microbiome under these conditions demonstrate the ability of the refrigeration to slow the decay process. This is in line with a study performed by Tuomisto et al. (2013) that used real-time qPCR to track six bacterial families in the stool of human autopsy cases finding no significant changes up to 5 days PM. A study that aimed to compare the thanatomicrobiome of internal organs of a rabbit model found no significant differences in the bacterial communities residing in the cecum up to 48 h PM when stored at 4°C (Lawrence et al., 2019). The bacterial communities present in the rectal cavity of the piglets showed inter-piglet variation reflecting observations in humans where the microbiome between individuals differs depending on a number of factors including genetics, age, diet, and environment (Hasan and Yang, 2019) and no trends were observed, likely due to our low sample size. The bacterial communities in P1 remained stable over the first 10 days PM reflecting results of our mouse study. In P2, although a change was observed between communities within the first 3 days there is arguably little difference observed over the remainder of the study period. The biggest shift was observed in Veillonellaceae, which are a bacterial family composed of anaerobic and microaerophilic bacteria. Their ability to thrive in such conditions explains their proliferation as time progresses. Microbial succession has been shown in various studies to be directly influenced by oxygen availability, with a longer PMI associated with a shift from aerobic to anaerobic bacteria at the completion of the bloat stage of decomposition (Hyde et al., 2013, 2015; Metcalf et al., 2013; Pechal et al., 2014; Lawrence et al., 2019).

We observed an exponential increase of Pseudomonadaceae members within the nasal cavity of the piglets as time PM increased. Studies have identified Pseudomonadaceae on porcine carcasses and are associated with food spoilage, given their ability to proliferate at low temperatures (Raposo et al., 2016). It is also interesting to note that in a recent study by Hurtado et al. (2018) who investigated the impact of PMI on microbiological results found an increase of Pseudomonas genus identification when using molecular analysis.

The mouse model used in this study failed to demonstrate ubiquitous PM translocation using 16S rRNA gene sequencing, with most samples possessing bacterial DNA loads too low to be sequenced. These results are reflected in a similar study performed on rabbit carcasses which investigated the effects of temperature on the thanatomicrobiome where they failed to amplify bacterial DNA in both lung and kidney samples due to low bacterial load (Lawrence et al., 2019). The study also obtained low sequencing reads for these visceral tissues compared to those obtained from the cecum and ileum and hypothesised that the low bacterial DNA present in these samples resulted in non-specific amplification of host DNA that then failed to align to the reference database (Lawrence et al., 2019).

The low-level detection of Enterobacteriaceae and Enterococcus in the mouse tissues and the late signal produced using a highly sensitive assay show that the bacteria are potentially present within the tissue, but at very low numbers. If this were due to translocation, it is unlikely that it would only be seen in these few tissues at seemingly random time points. This observation could therefore represent PM bacterial translocation but given the low read numbers this is unlikely. It has been found elsewhere that in human autopsies Enterobacteriaceae is found at higher numbers in autopsies performed more than 24 h after death using both molecular and conventional culture analysis (Hurtado et al., 2018). This finding is also in line with previous work that has shown poor correlation between post-mortem interval (PMI) and positive bacterial cultures in clinical autopsy investigation (Lobmaier et al., 2009; Weber et al., 2010).

We found a larger number of positive tissues and sequencing read numbers in the piglet model compared to the mouse model. The similarity of bacterial families shared between tissues collected from the same piglets suggest that these bacterial families originate from the piglet itself, rather than the external environment. Given the lack of concordance between bacterial families found in the tissues and swabs it is unlikely that these bacteria originate from such sites. Bacterial families, such as Moraxellaceae, Clostridiaceae, Flavobacteriaceae and Peptostreptococcaceae have been found in high abundance on the skin, tonsil, and nasal cavities of piglets in various studies (Lowe et al., 2012; McIntyre et al., 2016; Strube et al., 2018). It is therefore possible that these positive tissues have arisen from these sites as a result of our sampling technique.

The vast number of positive cultures that are typically detected in human SUDI PM examinations are reflected in positive piglet tissues in this study; > 80% of human PM tissues from SUDI autopsies generate positive culture results (Weber et al., 2010; Pryce et al., 2011a). In these human tissues a majority of bacteria identified are enteric bacteria (Highet, 2008), which differs from the bacterial species identified in this study. This may be due to differences in the PM translocation process or perhaps contamination occurs at a higher rate during human autopsy and tissue processing. The technique used in this study could also introduce differences in results as for a positive bacterial culture only a single viable bacteria needs to be present within the sample. Examining bacterial components using molecular targets, such as the 16S rRNA gene allow a more accurate evaluation of bacterial communities.

We acknowledge limitations of this study including the lack of skin sampling which could enhance the interpretation of bacteria within tissues. It is also important that this study was carried out on healthy animals free of known infection. If an infection were to be present in these animals the conclusions may have been different, perhaps due to increased invasive abilities of pathogenic bacteria, their disruption to commensal microbes or damage to tissue epithelium compromising barrier function. We also acknowledge that the time between death and sampling in these model organisms was also controlled as would not be the case in SUDI. Although this study could not detect PM bacterial translocation in these model organisms, it is important to note that the PM process in human subjects may differ. Given the small sample size and the use of animal models, no clinical conclusions can be made but this study provides an insight into the investigation of PM overgrowth of bacteria and acts as a proof-of-principle study into the use of 16S rRNA gene sequencing under these circumstances.

A deeper understanding of the bacterial changes that occur PM in clinical settings will allow for improved diagnostics, particularly in cases of SUDI due to sub-clinical infection. By determining the PM process using 16S rRNA gene sequencing we have demonstrated how this technique could be incorporated into routine PM diagnosis of infection. The results of this study did not, however, provide convincing evidence to support ubiquitous PM bacterial translocation. Future works should focus on implementing this technique on PM tissues from cases of SUDI to enhance our understanding and retrospective diagnosis of fatal infection.

## Data Availability Statement

The datasets presented in this study can be found in online repositories. The names of the repository/repositories and accession number(s) can be found below: NCBI BioProject PRJNA698635.

## Ethics Statement

Ethical review and approval was not required for the animal study as animals whose carcasses were used in this study were not killed for this work. No aspect of the animal’s life or death was altered for the scientific purpose of the present study.

## Author Contributions

NS and NK came up with the conceptual idea for this study and the clinical need for such work. LG and DA devised the study plan. LG performed all the experimental work, developed bioinformatics pipelines for the data analysis and visualisation, and wrote the manuscript with consultation from NS, NK, and DA. All authors contributed to the article and approved the submitted version.

## Conflict of Interest

The authors declare that the research was conducted in the absence of any commercial or financial relationships that could be construed as a potential conflict of interest.
