# Supplementary material for: Characterising Post-mortem Bacterial Translocation Under Clinical Conditions Using 16S rRNA Gene Sequencing in Two Animal Models
Source: Front Microbiol. 2021 May 31;12:649312. doi: 10.3389/fmicb.2021.649312 (PMC8200633; doi:10.3389/fmicb.2021.649312)
Supplement: Supplementary file 1 [file Data_Sheet_1.pdf]

Supplementary methods 1

Piglet tissue identity confirmation

Tissue-specific RT-PCRs were performed to confirm the tissue type obtained at sampling. Differentially expressed mRNA was identified using the University of California Santa Cruz (UCSC) Genome Browser (1) followed by primer design using the NCBI Primer-BLAST tool. Target genes were as follows: APOC4 (liver) (forward primer (FP): 5'-TGG TTC CGG GCA AGA TGA AG-3', reverse primer (RP): 5'-GCT ATG GGC CTT GTT CAG GA-3'), ANKRD1 (heart) (FP: 5'-TGC TCG GGA TAA GTT GCT CA-3', RP: 5'-GGC GCC ATA CGT AAT CAG GA-3'), SFTPC (lung) (FP: 5'-GGT CTA TGA CTA CCA GCG GC-3', RP: 5'-CAG CTT AGA GGT AGG CGT CG-3'), GAPDH (control) FP: 5'-ACA CTC ACT CTT CTA CCT TTG-3', RP: 5'-CAA ATT CAT TGT CGT ACC AG-3')

The reverse transcriptase reaction was carried out using the ProtoScript II First Strand cDNA synthesis kit (New England Biolabs) according to the manufacturer's instructions. Briefly, to remove secondary structure 5μL of total RNA was added to 2μL of oligo d(T) 23 VN oligonucleotide and made up to a total reaction volume of 8μL using 1μL of nuclease free water and kept at 65°C for 5 minutes. To complete the reaction 10μL of ProtoScript II reaction mix and 2μL of ProtoScript II Enzyme mix were added to each sample and incubated at 25°C for 5 minutes followed by 42°C for 90 minutes and a final 5 minutes at 80°C. No-RT control reactions consisting of all components of the reaction without the reverse transcriptase enzyme were used to monitor potential products arising from genomic DNA.

The qPCR reaction was performed as follows: 10μL Bioline SensiFAST SYBR No-ROX master mix (Bioline), 0.5μM forward primer, 0.5μM reverse primer, 2μL cDNA template and 6μL nuclease free water to make a final volume of 20μL. The reactions were cycled on a BioRad CFX96 real time system with the following cycling parameters: an initial 3 minutes at 95°C followed by 45 cycles of 95°C for 5 seconds and 65°C for 30 seconds. This was followed by the addition of a melt curve to monitor non-specific amplicons. Each reaction contained a positive control and a no template control (NTC). Results were analysed using BioRad CFX Manager (version 3.1).
