# Supplementary material for: Characterising Post-mortem Bacterial Translocation Under Clinical Conditions Using 16S rRNA Gene Sequencing in Two Animal Models
Source: Front Microbiol. 2021 May 31;12:649312. doi: 10.3389/fmicb.2021.649312 (PMC8200633; doi:10.3389/fmicb.2021.649312)
Supplement: Supplementary file 2 [file Data_Sheet_2.pdf]

Supplementary methods 2

Enterobacteriaceae probe-based qPCR

DNA extracted from mouse tissue was amplified using the QuantiTect Multiplex PCR Mastermix (Qiagen). The reaction mixture was as follows: 14μL QuaniTect 2x mastermix, 0.36μM Enterobacteriaceae forward primer (FP) (5’ ACC TGG GTA CWA CCA AC 3’), 0.36μM reverse primer (RP) (5’ GTC ACT GCC TGA CGT TTA 3’) and 0.36μM probe (5’ FAM-AGG ATG GTG AAA CTC TGG TWG GTC AGC C – BHQ-1 3’) and 10μL DNA extract. Nuclease-free water was used to make the total reaction volume to 28μL Mouse GI tract samples that were positive for Enterobacteriaceae served as positive controls and a no-template control consisting of nuclease-free water. DNA extraction controls were also included. Reactions were cycled on a thermocycler with the following cycling conditions: initial heat activation at 95°C for 15 minutes, followed by 45 cycles of denaturation at 94°C for 15 seconds, annealing at 58°C for 30 seconds and extension at 72°C for 30 seconds. Results were analysed using the CFX Manager Software (BioRad).
