# Supplementary material for: Characterising Post-mortem Bacterial Translocation Under Clinical Conditions Using 16S rRNA Gene Sequencing in Two Animal Models
Source: Front Microbiol. 2021 May 31;12:649312. doi: 10.3389/fmicb.2021.649312 (PMC8200633; doi:10.3389/fmicb.2021.649312)
Supplement: Supplementary file 3 [file Data_Sheet_3.pdf]

Supplementary methods 3

Enterococcus qPCR

The Enterococcus qPCR was carried out using QuantiTect SYBR-green Mastermix. The reaction mixture consisted of 12.5µL QuantiText 2x mastermix, 0.4µM FP (5’ CGT CGC AAG MMC AAA GAG 3’), 0.4µM RP (5’ TTA CCG CGG CTG CTG GCA C 3’), 2µL DNA extract and nuclease-free water to reach a final reaction volume of 25µL. Mouse GI tract samples that were positive for Enterococcus served as positive controls and a no-template control consisting of nuclease-free water as negative controls. DNA extraction controls were also included. Reactions were cycled on a thermocycler with the following cycling conditions: initial heat activation at 95°C for 15 minutes, 45 cycles of denaturation at 94°C for 15 seconds, annealing at 58°C for 30 seconds and extension at 72°C for 30 seconds, followed by a melt curve. Results were analysed using the CFX Manager Software (BioRad).
