# Supplementary material for: Characterising Post-mortem Bacterial Translocation Under Clinical Conditions Using 16S rRNA Gene Sequencing in Two Animal Models
Source: Front Microbiol. 2021 May 31;12:649312. doi: 10.3389/fmicb.2021.649312 (PMC8200633; doi:10.3389/fmicb.2021.649312)
Supplement: Supplementary file 4 [file Image_1.pdf]

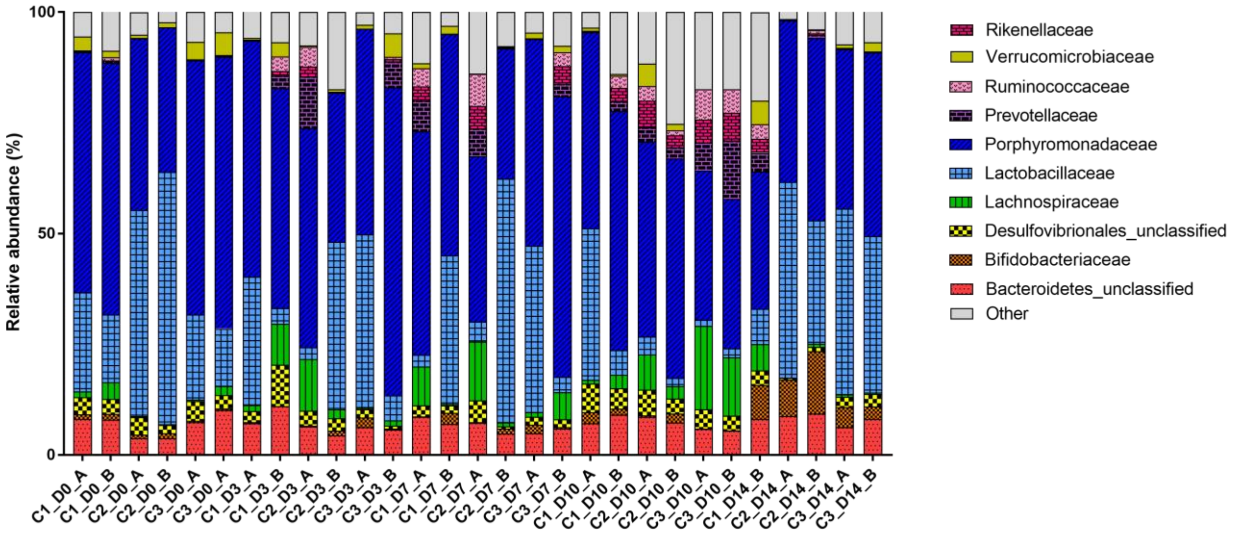

Supplementary figure 1

Relative abundance of the top 10 bacterial families in all mouse gastrointestinal (GI) tract samples collected over the 14-day study period. The x-axis describes the cage from which the sample was collected (C1, C2, C3) and the day on which the sample was collected (D0, D3, D7, D10, D14). Samples A and B are duplicate samples collected from the same mouse.
