# Supplementary material for: Characterising Post-mortem Bacterial Translocation Under Clinical Conditions Using 16S rRNA Gene Sequencing in Two Animal Models
Source: Front Microbiol. 2021 May 31;12:649312. doi: 10.3389/fmicb.2021.649312 (PMC8200633; doi:10.3389/fmicb.2021.649312)
Supplement: Supplementary file 5 [file Image_2.pdf]

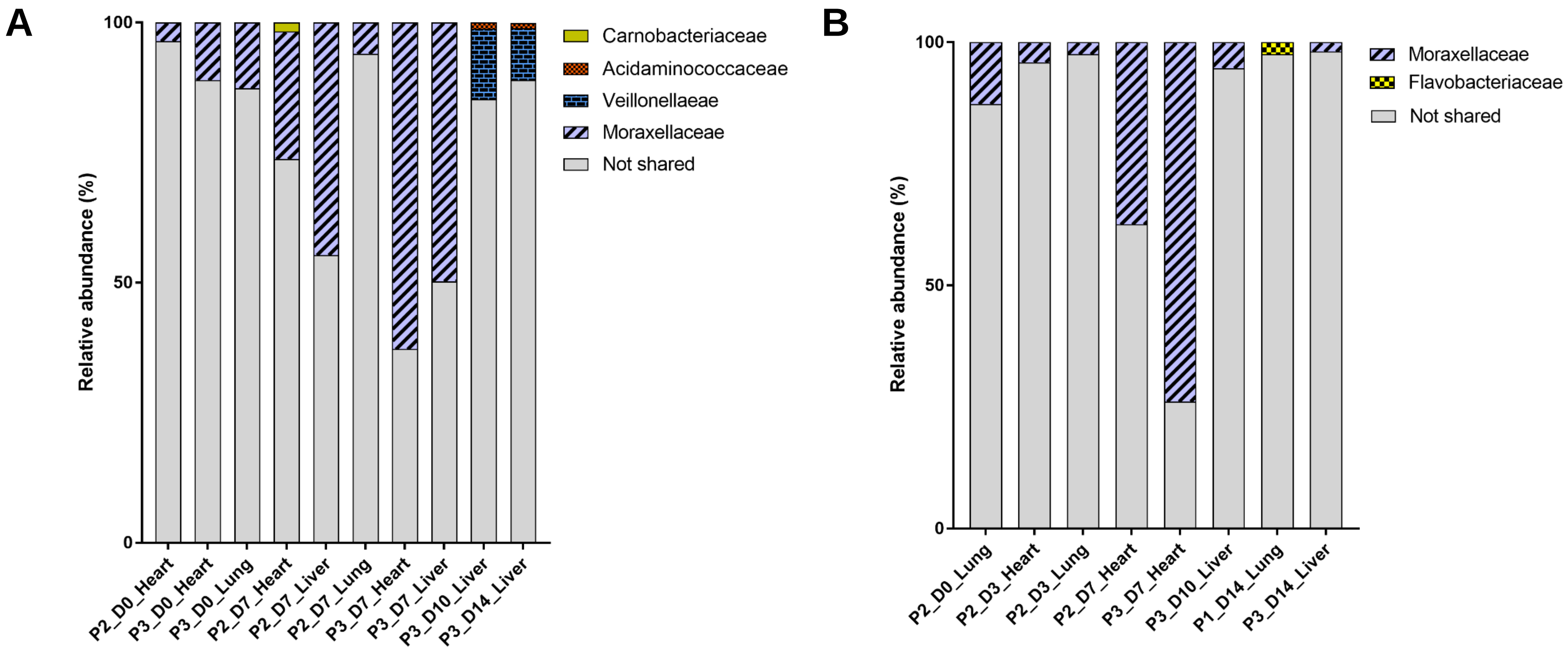

Supplementary figure 2

Bacterial families that piglet tissue samples shared with (A) rectal swabs, and (B) nasal swabs collected from the same piglet.
