# Supplementary material for: Characterising Post-mortem Bacterial Translocation Under Clinical Conditions Using 16S rRNA Gene Sequencing in Two Animal Models
Source: Front Microbiol. 2021 May 31;12:649312. doi: 10.3389/fmicb.2021.649312 (PMC8200633; doi:10.3389/fmicb.2021.649312)
Supplement: Supplementary file 6 [file Table_1.pdf]

	D0			D3			D7			D10			D14		
	C1	C2	C3	C1	C2	C3	C1	C2	C3	C1	C2	C3	C1	C2	C3
Blood	N	N	N	N	N	N	N	N	N	N	N	N	N	**P**	N
Heart	N	N	N	N	N	N	N	N	N	N	**P**	N	N	N	N
Liver	N	N	N	**P**	**P**	N	N	N	N	N	N	N	N	N	N
Lung	N	N	N	N	N	N	N	N	N	N	N	N	N	N	N
Spleen	N	N	N	N	N	N	N	N	N	N	N	N	N	N	N

Supplementary Table 1

Mouse tissues positive for the 16S rRNA gene. Table headers describe the cage from which the sample was collected (C1, C2, C3) and the day on which the sample was collected (D0, D3, D7, D10, D14). P represents tissue samples positive for 16S rRNA gene following sequencing and quality filtering and N represents negative for 16S rRNA gene after sequencing and quality filtering.
